# Diagnostic potential of near‐infrared spectroscopy in mild cognitive impairment and neurodegenerative disorders: Implications for resource‐limited settings

**DOI:** 10.1002/alz.70769

**Published:** 2025-10-08

**Authors:** George Nkrumah Osei, Ansumana Bockarie, Albert Akpalu, Peter Osei‐Wusu Adueming, Maxwell Hubert Antwi, Foster Appiah, Linus Kpelle, David Mawutor Donkor, David Larbi Simpong

**Affiliations:** ^1^ Department of Medical Laboratory Science School of Allied Health Sciences University of Cape Coast Cape Coast Ghana; ^2^ Department of Internal Medicine and Therapeutics, School of Medical Sciences University of Cape Coast Cape Coast Ghana; ^3^ Department of Medicine University of Ghana Medical School Accra Ghana; ^4^ Laser and Fibre Optics Centre, School of Physical Sciences University of Cape Coast Cape Coast Ghana; ^5^ Department of Medical Laboratory Science, Faculty of Health Sciences Koforidua Technical University Koforidua Ghana

**Keywords:** Alzheimer's disease, functional connectivity, mild cognitive impairment, near‐infrared spectroscopy, neurodegenerative diseases, oxyhemoglobin

## Abstract

**BACKGROUND:**

Near‐infrared spectroscopy (NIRS) is emerging as a promising tool for early detection of mild cognitive impairment (MCI) and neurodegenerative diseases, especially where advanced imaging is limited.

**METHODOLOGY:**

This systematic review investigates NIRS's diagnostic capabilities. Adhering to Preferred Reporting Items for Systematic Reviews and Meta‐Analyses guidelines, we conducted a comprehensive review of studies using NIRS for cognitive assessment in MCI and neurodegenerative conditions.

**RESULTS/DISCUSSION:**

NIRS effectively assesses cognitive function, identifying reduced prefrontal connectivity in MCI and subjective cognitive decline (SCD). Interestingly, while SCD patients maintain stronger brain network integrity, NIRS reveals decreased oxyhemoglobin levels in Alzheimer's disease (AD) patients’ dorsolateral prefrontal cortex. Combining NIRS with graph analysis, cognitive tasks, and machine learning significantly boosts diagnostic accuracy. Moreover, NIRS can differentiate between neurodegenerative disorders and, with concurrent electroencephalography, offers enhanced understanding of brain connectivity issues in AD. Our findings emphasize NIRS's considerable potential to improve cognitive assessment and neurodegeneration diagnosis.

**Highlights:**

- Mild cognitive impairment (MCI) individuals show disruptions in neurovascular coupling and functional connectivity, particularly in the dorsolateral prefrontal cortex during near‐infrared spectroscopy (NIRS) assessments.
- Significant reductions in oxyhemoglobin (HbO_2_) levels are observed in the dorsolateral prefrontal cortex of amnestic MCI (aMCI) individuals compared to healthy controls.
- Individuals with subjective cognitive decline (SCD) show lower HbO_2_ levels than healthy individuals, while aMCI individuals show even more pronounced reductions.
- The dorsolateral prefrontal cortex is identified as a critical area for Alzheimer's disease (AD) assessment using NIRS, correlating with cognitive performance.
- Differences in Broca's area activation during language tasks help distinguish behavioral variant frontotemporal dementia from AD, revealing unique cognitive profiles through NIRS.

## 1 BACKGROUND

Neurodegenerative diseases (NDDs) are a group of progressive and debilitating neurological disorders characterized by the gradual loss of neuronal structure and function, resulting in cognitive, motor, and behavioral impairments.^1^ While not a natural part of aging, these conditions result from complex gene–environment interactions that substantially influence an individual's susceptibility.^2^ NDDs, including Alzheimer's disease (AD), Parkinson's disease (PD), amyotrophic lateral sclerosis (ALS), Lewy body dementia (LBD), and frontotemporal dementia (FTD), collectively affect > 40 million individuals globally, representing a significant and growing public health concern.^3^ Mild cognitive impairment (MCI), a clinical condition characterized by cognitive decline beyond normal aging yet without significant impact on daily functioning, serves as an intermediate stage between normal cognition and several NDDs.^4^ However, detecting MCI remains challenging due to high misclassification rates. Cerebrospinal fluid (CSF) biomarkers have a 24% MCI misclassification rate, while traditional screening tools such as the Mini‐Mental State Examination (MMSE) and the Montreal Cognitive Assessment (MoCA) are culturally insensitive, leading to diagnostic limitations.^5^, ^6^, ^7^

AD, the most prevalent NDD, remains without cure and continues to impose a growing medical and socio‐economic burden. Its pathogenesis is multifactorial, with neural alterations occurring at nearly every cellular level.^8^ Clinically, AD is primarily marked by impaired recent memory, which progressively leads to deficits in other cognitive functions, including speech, orientation, judgment, executive function, and behavioral regulation, ultimately affecting daily activities.^9^ Research on the global impact of AD suggests that early detection and treatment of cognitive impairment in its initial stages can delay AD onset by 5 years.^10^ This delay could lead to a nearly 57% reduction in dementia cases while also cutting annual medical insurance costs by almost half. Beyond AD, FTD is associated with atrophy of the frontal and temporal lobes, while dementia with Lewy bodies is characterized by the presence of Lewy body inclusions and accompanying motor disturbances. PD primarily affects motor control but also leads to significant cognitive changes over time.^11^, ^12^

Several brain diagnostic platforms for neurodegeneration detection and cognitive decline have surfaced, and over the past two decades, near‐infrared spectroscopy (NIRS) has revolutionized neuroscience, providing new ways to study human brain function.^13^ This technology offers a balance of moderate temporal resolution and fine spatial resolution, making it a powerful tool for studying brain activity and circulatory dynamics with precision. NIRS enables brain imaging by detecting differences in the absorption spectra of oxyhemoglobin (HbO_2_) and deoxyhemoglobin (HbR) as near‐infrared light passes through the cerebral cortex.^14^ This technique reflects hemodynamic responses triggered by neuronal activity, relying on the principle of neurovascular coupling. An increase in blood flow to a region of neural activation leads to a rise in HbO_2_ concentration while HbR levels decrease due to the wash‐out effect of blood circulation, collectively serving as an indirect marker of brain cell activation.^15^, ^16^

Magnetic resonance imaging (MRI) and computed tomography (CT) are strongly recommended for the early evaluation of NDDs, while positron emission tomography (PET) and single photon emission computed tomography (SPECT) are used for more complex cases. However, these imaging methods face challenges such as radiation safety concerns, the need for highly trained personnel, and the high costs of investment and maintenance, which limit their widespread use.^17^ NIRS has demonstrated strong consistency with MRI, PET, and electroencephalography (EEG) in brain network studies while offering several distinct advantages. NIRS measurements of these hemoglobin responses have been found to align spatially and temporally with the blood oxygen level–dependent signal detected by MRI.^16^ NIRS also provides superior spatial resolution and is less susceptible to motion artifacts compared to EEG. Additionally, it offers better temporal resolution than MRI and PET, allowing precise detection of hemodynamic changes for continuous real‐time monitoring of the cerebral cortex.^10^, ^18^ Unlike MRI, NIRS is compatible with individuals who have metal accessories or implants, does not require confinement in a small space reducing the risk of claustrophobia, and does not produce disruptive noise.^10^, ^18^ Compared to PET, NIRS is more portable, eliminates the risk of ionizing radiation exposure, and is significantly more cost effective. Moreover, unlike EEG, NIRS does not require a conductive medium, enhancing user comfort.^10^

Over the past few decades, there has been a growing interest in using NIRS for cognitive assessment, particularly in the early detection of NDDs. For NDD diagnosis in resource‐constrained settings, NIRS stands out as a potential game changer. This portable and cost‐effective neuroimaging tool overcomes some of the limitations of costly MRI and PET scans often unavailable in resource‐constrained settings. Because NIRS is non‐invasive and safe, it enables frequent monitoring of dementia progression and treatment without patient discomfort or radiation. Its user‐friendly nature and portability mean it can be widely applied in various clinical and community environments, including hard‐to‐reach rural areas. Furthermore, NIRS's capacity to detect hemodynamic brain changes provides critical early diagnostic information on vascular and metabolic dysfunctions. Despite promising advancements, existing studies remain heterogeneous in terms of design, population characteristics, and research methodologies, creating challenges in synthesizing evidence and drawing definitive conclusions. This review aims to address these gaps by systematically evaluating how NIRS has been used in assessing cognitive decline related to MCI and various NDDs. By identifying patterns, methodological variations, and emerging trends, this study highlights the potential of NIRS as a reliable tool for improving diagnostic accuracy and efficiency. Furthermore, it lays a foundation for future investigations into the use of NIRS in diagnosing conditions such as MCI, AD, and PD, ultimately contributing to the advancement of early intervention strategies and precision medicine in NDDs.[Fig alz70769-fig-0001]

## 2 METHODOLOGY

This systematic review was conducted following the Preferred Reporting Items for Systematic Reviews and Meta‐Analyses (PRISMA) guidelines. The methodology was registered in PROSPERO (ID: CRD42025634879) before initiating the literature search.^19^ A comprehensive search was performed across PubMed, Scopus, and Google Scholar databases until May 2025 using keywords such as “cognitive impairment,” “neurodegenerative disease,” “Alzheimer's disease,” “diagnosis,” “detection,” and “near‐infrared spectroscopy” to ensure a focused selection of studies relevant to the application of NIRS in MCI and NDD diagnosis and monitoring. A detailed search strategy is provided as supplementary material in supporting information.

Studies that examined human subjects with cognitive impairment or neurodegenerative disorders using NIRS as a diagnostic or investigative tool were included. Eligible studies comprised original research, including clinical trials, cohort, case–control, and cross‐sectional studies that evaluated the effectiveness, accuracy, and reliability of NIRS in detecting cognitive impairment or NDDs. Studies were excluded if they used animal models, involved in vitro procedures, were not published in English, did not focus on NIRS, or lacked relevant outcome data. The exclusion of studies published in non‐English languages was due to challenges in accurately interpreting and critically appraising them, as well as limited resources for translation. To ensure relevance and incorporate the latest advancements in neuroimaging technologies, the review focused on studies published from 2015 onward. Filtering results to this timeframe allowed for a more up‐to‐date assessment of NIRS in cognitive assessment. Additionally, reference lists of the included studies were examined to identify further relevant publications from the same period.

**FIGURE 1 alz70769-fig-0001:**
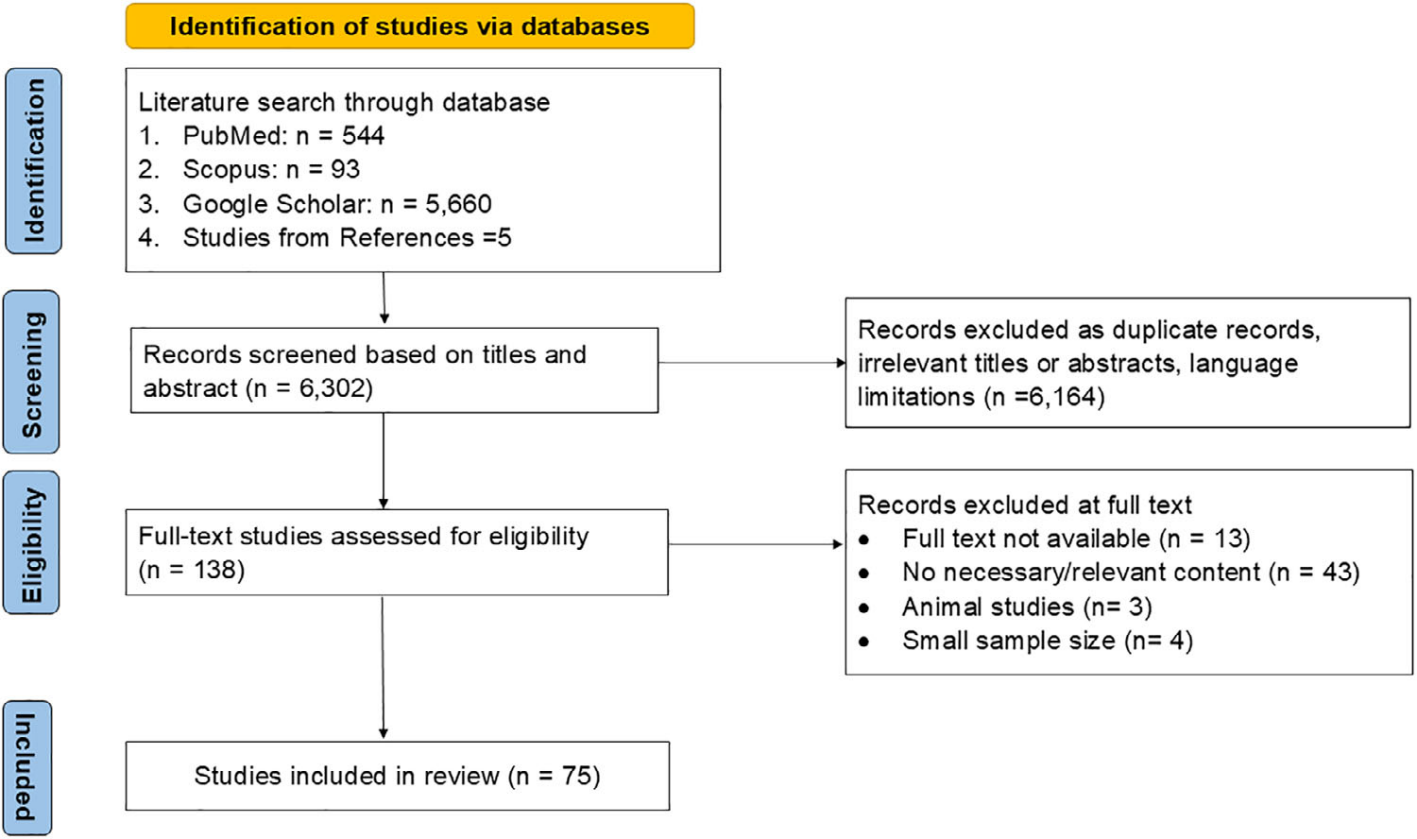
Preferred Reporting Items for Systematic Reviews and Meta‐Analyses flow diagram of the screening and selection of studies.

Data extraction and synthesis were conducted systematically. Two independent reviewers screened titles and abstracts for eligibility, with ambiguous studies reviewed by a third investigator. Full‐text assessments were conducted in the second stage, and a third reviewer resolved uncertainties. The PRISMA flowchart documents the selection process (Figure 1). Extracted data included study design, sample size, population characteristics, NIRS techniques, type of neurocognitive disorder, and key findings (Table 1). Findings of studies were summarized using a narrative synthesis due to expected heterogeneity precluding quantitative pooling. The expected sources of heterogeneity included clinical differences (variations in participant characteristics, interventions, or outcomes), methodological differences (study design variations), and statistical differences.

**TABLE 1:** Representative summary of included studies.

No.	Study	Study design	Country of study	Sample size	Types of techniques used	Type of neurocognitive disorder	Key findings
1	Pu et al., 2025	Prospective, cross‐sectional study	China	246 participants (55 normal controls, 80 SCD patients, and 111 MCI patients)	fNIRS and machine learning	SCD and MCI	Analysis of activated PFC during cognitive scale performance, combined with subject‐wise cross‐validation, revealed that as cognitive function declines, prefrontal connectivity becomes less stable. Additionally, individuals with SCD and MCI exhibit reduced communication between the left and right sides of the prefrontal cortex.
2	Tso‐Yen et al., 2024	Prospective, cross‐sectional study	Taiwan	205 healthy and MCI participants	fNIRS and GBIT	MCI	Cognitive performance assessed using MMSE revealed a noticeable decline with age, highlighting age‐related cognitive decline. Additionally, HbO_2_ and HbR patterns observed during the 3 minute GBIT test varied with age, especially in individuals > 75 years, suggesting changes in neural activity over time.
3	Petrillo et al., 2025	Prospective, cross‐sectional study	United States	83 participants (17 HCs, 37 cognitively normal, 29 MCI)	fNIRS, dual‐tasking	MCI	Cognitively healthy individuals exhibited higher complexity in brain activity signals while older individuals with cognitive impairment exhibit reduced signal complexity, reflecting declines in neural processing efficiency.
4	Ateş et al., 2017	Case–control study	Turkey	246 participants (20 normal controls, 20 AD patients)	fNIRS, cognitive tasks	AD	Individuals with AD showed increased activity in the left ventral PFC during the positive emotional words condition compared to HCs. Similarly, heightened activation was observed in the right ventrolateral PFC in AD patients during the neutral words condition.
5	Mol et al., 2021	Case–control study	Switzerland and Netherlands	202 participants (108 cognitively normal, 37 MCI, and 57 AD)	NIRS and TCD	MCI, AD	Varying correlations between NIRS‐ and TCD‐derived cerebral autoregulation measures were observed, with low correlations (0.22–0.30) during supine rest and stronger correlations (0.46–0.61) during repeated sit‐to‐stand transitions in cognitively impaired patients.
6	Herrmann et al., 2024	Prospective, observational, long‐term follow‐up study	Germany	471 participants (424 healthy and 47 MCI)	fNIRS and VSEP	MCI	NIRS analysis during verbal fluency and visuospatial tasks showed lower HbO_2_ levels in the frontotemporal cortex of cognitively impaired individuals. Additionally, VSEP revealed prolonged latencies in cognitively impaired individuals.
7	Vermeij et al., 2017	Interventional, longitudinal study	Netherlands	35 participants (21 healthy older adults and 14 MCI patients)	fNIRS	MCI	MCI patients exhibited maximum HbO_2_ increase at lower WM load compared to healthy older adults, who showed peak activation at higher WM load (2‐back). However, the HbR response did not provide a clear pattern.
8	Talamonti et al., 2022	Longitudinal study	Canada	32 participants (24 HCs and 8 MCI patients)	NIRS, dual‐task walking paradigm	MCI	Regular physical activity was associated with improved cognitive performance and more efficient brain oxygenation over 1 year, with a stronger impact observed in individuals with MCI, though benefits were also evident in healthy adults.
9	Xu et al., 2024	Case–control study	China	40 participants (21 older adults diagnosed with MCI and 19 healthy older adults)	fNIRS	MCI	Older adults with MCI demonstrated significantly lower balance control than healthy older adults, especially under dual‐task conditions. Additionally, individuals with MCI exhibited higher PFC activation, suggesting increased neural effort to compensate for balance deficits.
10	Z. Yang et al., 2025	Case–control study	China	24 participants (17 AD patients and 7 HCs)	fNIRS, Verbal Fluency Task	AD	Significant differences in cerebral hemodynamic responses between AD patients and the control group were found, with AD patients exhibiting decreased HbO_2_ concentrations. The decline in FC was primarily observed in the dorsolateral PFC and other brain regions.
11	Metzger et al., 2016	Case–control study	Germany	24 participants (8 AD patients, 8 bvFTD patients, and 8 HCs)	fNIRS, Verbal Fluency Task	AD and bvFTD	AD patients exhibit a significantly weaker activation pattern in key brain regions, including the dorsolateral PFC, supplementary motor area, and Wernicke's area during the Verbal Fluency Task, particularly in the left hemisphere. In contrast, bvFTD patients display distinct activation variations, characterized by increased activity in Broca's area.
12	J. Kim et al., 2022	Prospective, interventional, short‐term longitudinal study	Republic of Korea	97 participants (28 cognitively normal, 32 preclinical AD, 21 MCI, and 16 AD)	Olfactory‐stimulation fNIRS	AD, MCI	Significant differences in oxygenation levels in the orbitofrontal cortex were observed, with reductions in patients with cognitive impairment, including mild AD and MCI, becoming more pronounced as cognitive severity increases.
13	J. Kim et al., 2023	Post hoc and prospective diagnostic pilot trial	Republic of Korea	133 participants (55 cognitively normal, 41 MCI, and 21 AD)	Olfactory‐stimulation fNIRS, machine learning	AD, MCI	Machine learning models leveraging olfactory‐stimulated oxygenation differences in the orbitofrontal cortex through fNIRS demonstrated superior accuracy in diagnosing MCI and AD.
14	Ferdinando et al., 2023	Case–control study	Finland	22 participants (8 AD and 14 age‐matched healthy subjects)	MRI‐compatible novel multiwavelength fNIRS	AD	In the cardiac band, healthy individuals exhibit greater time‐domain lag variation in HbR‐CSF, dHbR‐CSF, HbR‐dCSF, and dHbR‐dCSF pairs compared to AD patients. Additionally, venous HbR blood signal coupling with CSF is more variable in healthy individuals, whereas in AD patients, it becomes more uniform. Under fast sampling conditions with minimal overlapping windows, the HbR‐CSF coupling in AD patients gradually shifts to a monotonous pattern over time.
15	Mızrak et al., 2024	Observational case–control study	Turkey	30 participants (14 AD and 16 age‐matched healthy subjects)	fNIRS, Verbal Fluency Task	AD	Increased interhemispheric connectivity was found in HCs compared to AD patients. AD patients exhibit persistent functional hemispheric asymmetry.
16	Q. Li et al., 2024	Short‐term longitudinal experimental study	China	97 healthy and cognitively impaired participants	fNIRS	Cognitive Impairment	Open skill exercises significantly enhance cognitive inhibition, leading to improved attention capacity, self‐regulation, and adaptability to environmental changes. In contrast, closed skill exercises are more effective in strengthening working memory, optimizing information processing, and enhancing storage capacity within cognitive functions.
17	Oyama et al., 2018	Cross‐sectional design	Japan	202 participants (108 normal, 55 MCI, and 39 severe cognitive impairment)	TRS	MCI	A significant positive correlation between MMSE and SO_2_ in the left PFC (*r* = 0.38, *P* < 0.01) and the right PFC was observed.
18	T. lok Lee et al., 2024	Cross‐sectional study design	Hong Kong	151 participants (37 with normal cognition, 86 with SMC [29 with severe SMC], 15 with aMCI, and 13 with naMCI)	fNIRS	MCI, SMC	Individuals with aMCI exhibited significantly lower levels of HbO_2_ compared to those with normal cognition, while severe SMC also correlated with reduced HbO_2_ levels.
19	Owens et al., 2024	Cross‐sectional observational study	United States	40 participants (20 MCI and 20 age‐matched HCs)	fNIRS	MCI	Individuals with MCI exhibit a significant decline in neurovascular coupling within the left dorsolateral PFC compared to cognitively normal individuals. Additionally, FC in the left dorsolateral PFC is significantly reduced in MCI.
20	Ung et al., 2020	Cross‐sectional study	Malaysia	61 participants (31 age‐matched HCs, 12 patients with MCI, and 18 mild AD)	fNIRS, visuospatial working memory task	MCI, mild AD	Patients with MCI demonstrated hyperactivation in the PFC at moderate task loads (HbO levels increased significantly). As task difficulty increased, the MCI group showed significant increases in HbO levels, while the AD group maintained static levels.
21	Y. L. Chan et al., 2020	Case–control study	Malaysia	42 participants (26 age‐matched HCs and 16 individuals diagnosed with mild AD)	fNIRS and OMSTs	Mild AD	Wavelet analysis for motion artifact correction with OMSTs for network binarization improves the reliability of FC assessments using fNIRS. This method highlights distinct connectivity patterns, showing that individuals in the mild AD group exhibited impaired FC.
22	Oyama & Sakatani, 2022	Observational cross‐sectional study	Japan	250 participants (115 patients with MCI and 135 HC)	Time‐resolved NIRS, machine learning	MCI	The deep neural network model using time‐resolved NIRS data demonstrated lower mean absolute error and mean absolute percentage error compared to predictions based on blood test data.
23	Yu et al., 2020	Cross‐sectional study	Republic of Korea	46 participants (23 HCs and 23 with MCI)	fNIRS	MCI	MCI group exhibited greater prefrontal functional connectivity compared to HCs, suggesting compensatory mechanisms.
24	E. Kim et al., 2021	Cross‐sectional study	Republic of Korea	60 participants (31 HCs, 11 patients with MCI, and 18 AD)	fNIRS	MCI, AD	The Delayed Matching‐to‐Sample task showed significantly higher HbO_2_ concentrations in the MCI group, whereas the Digit Span Task showed no significant differences.
25	Takahashi et al., 2022	Cross‐sectional study	Japan	132 participants (102 elderly individuals [61 with MCI and 41 without MCI] and 30 young controls)	NIRS	MCI	The finger‐tapping task appeared cognitively demanding for elderly individuals, including those with MCI, as indicated by sustained HbO_2_ levels.
26	Paraskevaidi et al., 2018	Case–control study	United Kingdom	284 participants (111 AD patients and 173 HCs)	NIRS	AD	Statistical analysis identified significant spectral differences between healthy controls and AD. Furthermore, multivariate classification models (PCA‐QDA) achieved high classification accuracy for AD individuals, with 92.8% accuracy, 87.5% sensitivity, and 96.1% specificity.
27	A. S. Chan et al., 2021	Pilot randomized controlled trial	Hong Kong	40 participants (18 MCI and 22 HCs)	fNIRS, photobiomodulation intervention	MCI	Photobiomodulation improved visual memory performance and reduced hemodynamic response during tasks in older adults with MCI.
28	C. Zhang et al., 2023	Prospective cross‐sectional study	China	127 participants (64 MCI and 63 HCs)	Multi‐dimensional fNIRS	MCI	3D time‐point HbO_2_ features provided the highest accuracy for detecting MCI, with 80.77% test accuracy, 76.92% sensitivity, and 83.33% precision. Additionally, HbO_2_ features were more effective than HbR features.
29	S. Park et al., 2024	Double‐blind, randomized controlled pilot study	Republic of Korea	21 MCI patients (10 participants inhaled saline [control], while the phytoncide group had 11 participants exposed to phytoncide)	fNIRS	MCI	The phytoncide group showed a significant improvement in Stroop task performance, while the saline group showed no significant change. Hemodynamic attenuation was observed in the left VLPFC of the phytoncide group.
30	Tian et al., 2022	Observational cross‐sectional study	China	56 participants (22 patients with MCI and 34 HCs)	fNIRS, Verbal Fluency Task	MCI	The HC group demonstrated significantly higher HbR concentrations in the left parietal lobule and left inferior parietal lobule during the category verbal fluency task compared to the MCI group
31	Salzman et al., 2023	Cross‐sectional pilot study	Canada	20 participants (10 SCD and 10 HCs)	fNIRS	SCD	Participants exhibited slower response times during dual‐task gait compared to single‐task condition. In non‐SCD group, gait speed was significantly slower in the dual‐task condition compared to the single task.
32	Perpetuini et al., 2019	Cross‐sectional study	Italy	22 participants (11 early AD and 11 HCs)	fNIRS, Clock Drawing Test, Digit Span Test, and Corsi Block Tapping Test	AD	The Clock Drawing Test effectively differentiated between AD patients and healthy controls, while the Digit Span Test and Corsi Block Tapping Test did not show significant distinctions between the two groups.
33	Perpetuini et al., 2020	Cross‐sectional study	Italy	35 participants (17 AD and 18 healthy controls)	fNIRS, EEG	AD	Conditional entropy EEG‐fNIRS metrics proved to be the most predictive across all tasks, particularly excelling during ROCF (recall), outperforming both EEG and fNIRS complexity metrics independently.
34	Teo et al., 2021	Cross‐sectional study	Australia	58 participants (26 healthy, 23 SMC, 9 dementia)	fNIRS	SMC, dementia	During single‐task gait, individuals with dementia showed a significantly higher HbO_2_ response compared to HCs and those with SMC, with no major differences between the latter two groups. However, in dual‐task gait, SMC participants exhibited an increase in HbO_2_, while those with dementia showed a decline.
35	Bjerkan et al., 2025	Case–control study	Slovenia	39 participants (19 AD and 20 HCs)	fNIRS, EEG, electrocardiography (ECG), and respiration effort analysis	AD	Phase coherence between instantaneous heart rate (IHR) and fNIRS signals was observed in the 0.052–0.6 Hz range, while respiration–fNIRS coherence occurred mostly in the 0.145–0.6 Hz range, with a consistent time lag of ≈ 2.5 seconds between oscillators. Notably, the AD group exhibited reduced IHR–fNIRS coherence across all channels
36	R. Li et al., 2018	Cross‐sectional study	China	30 participants (8 HCs and 9 MCI individuals, 6 mild AD, 7 moderate/severe AD)	fNIRS, Digit Verbal Span Task	MCI and AD	The HC group exhibited a typical response with a rapid increase in HbO_2_ concentration followed by a return to baseline, whereas the MCI group showed a delayed and mild rise, and AD groups demonstrated a noticeable decline and delay in activation.
37	D. Yang et al., 2020	Cross‐sectional study	Republic of Korea	24 participants (9 HCs and 15 MCI individuals).	fNIRS	MCI	MCI patients exhibited a marked reduction in HbO concentrations during the N‐back and Verbal Fluency Tasks (*P* < 0.001), whereas their response to the Stroop task remained similar to that of healthy controls (*P* = 0.06825).
38	Z. Wang et al., 2022	Cross‐sectional study	China	54 participants (38 HCs and 16 cognitive impaired individuals)	fNIRS	Cognitive impairment	Gait analysis revealed that step length in healthy participants was significantly longer than in cognitively impaired individuals (*P* < 0.01) during single‐task walking. In dual‐task walking, normal subjects exhibited greater step length, speed, and frequency than cognitively impaired individuals, with step length showing statistical significance (*P* < 0.05).
39	Ho et al., 2022	Cross‐sectional study	Republic of Korea	140 participants (53 age‐matched HCs and 83 asymptomatic AD, 50 prodromal AD, and 9 AD dementia)	fNIRS	AD	While brain activation remained similar in healthy individuals and AD patients during resting conditions, cognitive tasks revealed distinct differences, with prodromal AD patients exhibiting lower activation compared to HCs and asymptomatic AD.
40	J. H. Park, 2023	Cross‐sectional study	Republic of Korea	136 participants (84 HCs and 52 MCI)	fNIRS, Verbal Digit Span Task	MCI	fNIRS analysis revealed significantly reduced HbO levels in the left and right prefrontal cortex of MCI patients during cognitive tasks.
41	Z. Wang et al., 2024	Cross‐sectional study	China	33 participants (20 MCI and 13 SCD)	fNIRS, Verbal Fluency Tasks	MCI, SCD	FC analysis during resting‐state fNIRS revealed that SCD patients exhibited stronger FC patterns compared to MCI patients. During the verbal fluency task, SCD patients showed higher HbO_2_ concentration in the frontal lobe compared to MCI patients.
42	M. Kim et al., 2025	Cross‐sectional study	Republic of Korea	99 participants (54 HCs and 45 preclinical subjects who were amyloid beta positive)	fNIRS during a mixed phonemic and semantic verbal fluency task.	Preclinical AD	Preclinical AD individuals exhibited significantly higher interhemispheric FC of HbO_2_ during P1 compared to HC participants. Furthermore, a notable decline in interhemispheric FC of HbO_2_ was observed in the preclinical AD group between P1 and S1, whereas the HC group showed no such reduction.
43	Choi et al., 2024	Retrospective observational case–control study	Republic of Korea	28 participants (19 MCI patients and 9 HCs)	fNIRS	MCI	In the PFC, HbR activation levels gradually declined over time during Stroop task performance, with a notable reduction observed in the left dorsolateral PFC and frontopolar cortex in MCI patients at the 6 month follow‐up.
44	J. Wang et al., 2025	Case–control study	China	73 participants (19 patients with PD‐MCI, 21 with PD‐ NC, and 33 age‐matched HCs)	fNIRS during the Stroop Color‐Word Test	PD	The FC analysis revealed that patients with PD‐MCI exhibited significantly higher regional strength measures (S_l_, S_r_) and global efficiency compared to HC during the color‐word incongruent test. Additionally, RS_r_ demonstrated predictive value in distinguishing PD‐MCI from PD‐NC.
45	Ruan et al., 2025	Cross‐sectional study	China	53 participants (28 inpatients with AD and 25 with LBD)	fNIRS‐based verbal fluency task, blood biomarker analysis	AD, LBD	Compared to the LBD group, the mean HbO_2_ concentrations were significantly lower in the AD group in the left temporal cortex, right dorsolateral PFC, and right temporal cortex.
46	D. Yang et al., 2019	Cross‐sectional study	Republic of Korea	24 participants (15 MCI patients and 9 HCs)	fNIRS	MCI	Findings showed that the left PFC exhibited significantly higher HbO_2_ concentration changes in HCs compared to MCI patients across all tasks, with HCs displaying an earlier increase.
47	Keles et al., 2022	Case–control study	Turkey	39 participants (21 AD patients and 18 HCs)	fNIRS	AD	Resting‐state bilateral PFC activation was significantly reduced in AD patients compared to HCs, with the dorsolateral PFC demonstrating the highest relative activation in HC.
48	Tang & Chan, 2018	Cross‐sectional study	Malaysia	61 participants (18 mild AD patients, 12 MCI, and 31 normal aging individuals)	fNIRS	Mild AD, MCI	FC in normal aging was primarily concentrated in the left and middle PFC, shifting toward the right hemisphere in MCI and becoming evenly distributed in AD.
49	Bonilauri et al., 2022	Cross‐sectional study	Italy	39 participants (13 early PD patients and 26 moderate PD)	Whole‐head fNIRS	PD	Early PD patients showed increased activity in both left and right secondary visual cortices. In contrast, mild PD patients exhibited greater activation in the right PFC.
50	Uemura et al., 2016	Case‐control study	Japan	130 participants (64 older adults with aMCI and 66 cognitively HCs)	Multi‐channel fNIRS	aMCI	Subjects with aMCI showed reduced activation in the bilateral dorsolateral cortex during memory retrieval.
51	Marmarelis et al., 2017	Case–control study	United States	46 aMCI patients, 20 age‐matched controls [TCD group]; 43 aMCI patients, 22 age‐matched controls (NIRS group)	TCD, NIRS	aMCI	While dynamic cerebral autoregulation remained intact, MCI patients demonstrated significantly reduced vasomotor reactivity in response to CO_2_ changes, with more pronounced impairments in TOI/NIRS measurements.
52	Nakamura et al., 2021	Cross‐sectional observational study	Japan	63 participants (28 MCI and 35 non‐dementia control)	fNIRS, working memory tasks	MCI	The fNIRS index effectively differentiates MCI patients from HCs, showing high sensitivity (94%) and specificity (88%) for cognitive decline.
53	Greco et al., 2021	Case–control study	United States	129 participants (20 autopsy‐confirmed AD patients, 12 MCI patients, and 13 age‐matched controls)	NIRS	AD, MCI	NIRS can effectively differentiate AD and MCI patients from controls using specific spectral features at 860 and 895 nm.
54	Nguyen et al., 2019b	Cross‐sectional study	Republic of Korea	134 participants (42 with MCI and 53 cognitively HCs)	fNIRS with cognitive tasks	MCI	During the verbal fluency task, the HC group demonstrated significantly stronger inter‐hemispheric connectivity compared to intra‐hemispheric connectivity. In contrast, the MCI group showed no significant difference between inter‐ and intra‐hemispheric connectivity but exhibited lower inter‐hemispheric connectivity across multiple tasks.
55	Huang et al., 2024	Prospective cross‐sectional pilot study	Taiwan	131 participants (63 on‐stroke with normal cognitive function, 47 stroke with normal cognitive function, 6 non‐stroke with cognitive dysfunction, and 15 stroke with cognitive dysfunction)	fNIRS, Wisconsin Card Sorting Test	MCI	High‐scoring groups (cognitively normal) showed increased HbO_2_ and decreased deoxygenated hemoglobin, whereas low‐scoring groups (MCI) exhibited increases in both oxygenated hemoglobin and deoxygenated hemoglobin
56	Csipo et al., 2021	Prospective cross‐sectional observational study	United States	14 healthy young adults	fNIRS	MCI indirectly studied through cognitive workload responses	fNIRS assessments revealed increased HbO_2_ in the prefrontal cortex, particularly in the left dorsolateral PFC and motor cortex, during the 2‐back condition.
57	Katzorke et al., 2018	Cross‐sectional observational study	Germany	110 participants (55 healthy individuals and 55 MCI)	fNIRS during verbal fluency tasks	MCI	Individuals with MCI exhibited a significantly reduced hemodynamic response in the inferior frontotemporal cortex during the category verbal fluency task compared to HCs (*P* = 0.010).
58	R. Li, Rui, et al., 2019	Case–control study	China	32 participants (16 aMCI patients and 16 age‐ and education‐matched HCs)	fNIRS, graph‐based network metrics	aMCI	aMCI patients showed higher integration and segregation in brain networks compared to HCs, with significant network alterations in frontal, temporal, and parietal regions.
59	K. Lee et al., 2024	Randomized controlled pilot study	Republic of Korea	64 participants (43 adults with cognitive decline and 21 HCs)	fNIRS and machine learning‐based classification, photobiomodulation intervention	Cognitive decline	Significant cognitive improvements were observed in most metrics after transcranial photobiomodulation treatment, except for the Digit Span Test‐Backward and the Digit Symbol Coding test.
60	Mei et al., 2024	Cross‐sectional study	China	120 older adults (30 MCI, 28 LBD, 30 AD, and 32 cognitively normal individuals)	fNIRS	AD, LBD	The mean FC of the frontal and temporal lobe in resting state was significantly less in the AD (0.19 ± 0.11) group than in the MCI (0.23 ± 0.11), LBD (0.29 ± 0.12), and cognitively normal (0.40 ± 0.11) groups (*P* < 0.001).
61	Yoon et al., 201)	Observational cross‐sectional study	Republic of Korea	35 participants (20 normal elderly people and 15 patients with MCI)	fNIRS, two‐back test, Korean color word Stroop test, and semantic verbal fluency task	MCI	In the Stroop test, right‐sided hyperactivation was observed in the naMCI and normal groups, with the highest mean change in HbO_2_ in the naMCI group, followed by the normal group and the aMCI group.
62	D. Yang & Hong, 2021	Cross‐sectional study	Republic of Korea	24 subjects (15 MCI patients and 9 HC participants)	fNIRS, graph theory analysis, and traditional machine learning approach	MCI	The feature representation‐based transfer learning demonstrated improved accuracy in both the 30 second and 90 second cases, achieving 81.27% and 76.73%, respectively.
63	Yeung et al., 2016	Cross‐sectional study	Hong Kong	52 participants (26 older adults with MCI and 26 older adults with normal cognition)	fNIRS	MCI	The MCI group, unlike the normal cognition group, did not exhibit significantly increased frontal activations bilaterally when WM load increased. Compared to the normal cognition group, the MCI group had similar frontal activations at low load (*p* > 0.05 on all channels) but reduced activations at high load.
64	You et al., 2025	Cross‐sectional study	China	37 participants (18 individuals with MCI, and 19 healthy individuals)	fNIRS, upper limb multimodal tasks	MCI	Resting‐state brain activation and FC analyses revealed reduced activation and connectivity in MCI individuals, particularly in the left and right motor cortices and between frontal and motor regions.
65	Polak et al., 2017	A prospective, observational, long‐term follow‐up study	Germany	604 healthy, AD, and MCI participants	fNIRS and VSEP	AD, MCI	MCI was identified in 12.3% of participants, while AD patients exhibited reduced oxygenation levels, as measured by fNIRS, and both AD and MCI individuals showed significantly prolonged VSEP latencies.
66	Perpetuini et al., 2017	Case–control study	Italy	22 participants (11 healthy participants and 11 early AD patients)	fNIRS, free and cued selective reminding test	AD	Significant differences in multiscale entropy, measured using fNIRS, were observed between AD patients and HCs during the delayed free recall phase of the free and cued selective reminding test. These differences were specifically localized to Brodmann areas 9 and 46, which are regions of the brain associated with working memory functions.
67	S. Zhang et al., 2022	Cross‐sectional study	China	128 participants (64 patients with aMCI and 64 HCs)	fNIRS	aMCI	aMCI patients exhibited a marked reduction in FC throughout the brain, with notable impairments in the bilateral prefrontal, parietal, occipital, and right temporal lobes.
68	Sharon et al., 2020	Prospective case–control study	Israel	60 participants (34 patients with PD and 26 older adults)	fNIRS, obstacle negotiation task	PD	Prefrontal activation patterns in PD patients progressively increase across all phases of obstacle crossing, with significantly greater activation compared to healthy older adults, especially during and after the crossing phase.
69	J. H. Park, 2024	Prospective cross‐sectional study	Republic of Korea	230 participants (82 subjects with MCI and 148 HCs)	fNIRS, CNNs, 2‐back task	MCI	Significant differences in averaged HbO_2_ values between MCI and HC groups were found, and the CNN model could better discriminate MCI with over 89.57% accuracy than the Korean version of the Montreal Cognitive Assessment (MoCA).
70	Yoo et al., 2020	Prospective case–control study	Republic of Korea	26 participants (15 MCI and 11 HCs)	fNIRS, two‐back, Stroop, and semantic verbal fluency tasks	MCI	Region of interest selection identified key activated channels for each task, revealing that MCI patients exhibited no activation in the ventrolateral PFC compared to HCs.
71	Cicalese et al., 2020	Cross‐sectional study	China	29 participants (8 HCs, 8 MCI, and 8 patients with mild AD and 7 with moderate/severe AD)	EEG and fNIRS	MCI, AD	Hybrid EEG‐fNIRS model significantly outperforms unimodal EEG and fNIRS approaches in classifying subjects based on AD progression.
72	R. Li, Nguyen, et al., 2019	Case–control study	China	14 participants (8 HCs and patients with mild AD)	EEG and fNIRS	Mild AD	EEG‐fNIRS approach applied during a digit verbal span task reveals weaker cortical connectivity alterations associated in mild AD patients.
73	Yap et al., 2017	Case–control study	Malaysia	61 participants (31 HCs, 12 patients with MCI, and 18 patients with mild AD)	fNIRS, semantic verbal fluency task	MCI, mild AD	MCI exhibited a greater mean activation of both the right and left PFC, followed by HCs and mild AD. Analysis showed that in the left PFC, the time taken for HCs to achieve the activation level was shorter than MCI and mild AD.
74	Bu et al., 2019	Cross‐sectional study	China	54 participants (26 with MCI and 28 HCs)	fNIRS	MCI	The coupling strength of right PFC left PFC and left PFC to right occipital lobe as well as left PFC to left occipital lobe, right occipital lobe to left PFC and right PFC to left occipital lobe in MCI group were significantly lower than those in the control group.
75	C. Fan et al., 2025	Observational case–control study	China	136 participants (73 patients with MCI and 63 HCs)	fNIRS, Stroop test	MCI	Individuals with MCI and healthy controls exhibited increased cortical activation in the dorsolateral PFC, ventrolateral PFC, and parietal lobe while completing the Stroop task.

Abbreviations: AD, Alzheimer's disease; aMCI, amnestic mild cognitive impairment; bvFTD, behavioral‐variant frontotemporal dementia; CNN, convolutional neural network; CSF, cerebrospinal fluid; dCSF, first derivative of cerebrospinal fluid; dHbR, first derivative of deoxygenated hemoglobin; FC, functional connectivity; fNIRS, functional near‐infrared spectroscopy; EEG, electroencephalography; GBIT, Game‐Based Intelligence Test; HbO_2_, oxyhemoglobin; HbR, deoxyhemoglobin; HC, healthy control; LBD, Lewy body disease; MCI, mild cognitive impairment; MMSE, Mini‐Mental State Examination; MRI, magnetic resonance imaging; naMCI, non‐amnestic mild cognitive impairment; NIRS, near‐infrared spectroscopy; OMST, orthogonal minimal spanning tree; PD, Parkinson's disease; PD‐MCI, Parkinson's disease with mild cognitive impairment; PD‐NC, Parkinson's disease normal cognition; PFC, prefrontal cortex; ROCF, Rey–Osterrieth complex figure; SCD, subjective cognitive decline; SMC, subjective memory complaint; TCD, transcranial Doppler; TOI, tissue oxygenation index; TRS, time‐resolved near0infrared spectroscopy; VLPLC, ventrolateral prefrontal cortex; VSEP, vagus somatosensory evoked potential; WM, white matter.

Quality of included studies was evaluated using the Quality Assessment of Diagnostic Accuracy Studies‐2 (QUADAS‐2) tool, which assesses risk of bias and concerns regarding applicability across four key domains. The patient selection domain assesses whether participants were recruited appropriately and without bias such as avoiding inappropriate exclusions. The index test domain evaluates whether the diagnostic test under evaluation was conducted and interpreted without prior knowledge of the results of the reference standard. The reference standard domain evaluates whether the standard used to confirm the target condition was likely to correctly classify the condition, and whether its interpretation was performed independently of the index test. Finally, the flow and timing domain considers whether all participants received the same reference standard and whether the interval between the index test and reference standard was appropriate to avoid misclassification. A visual summary of these evaluations is presented in Figure S1 in supporting information. Studies deemed high quality demonstrated well‐defined patient selection, objective diagnostic testing, independent reference standard application, transparent timing, and minimal bias. Two reviewers independently assessed study quality, resolving discrepancies through discussion, with a third reviewer mediating when necessary. Because this systematic review relied on previously published, publicly accessible data, formal ethical approval was not required.

## 3 RESULTS AND DISCUSSION

The reviewed studies predominantly focused on populations with MCI (*n* = 49), followed by AD (*n* = 28), subjective cognitive decline (SCD)/subjective memory decline (SMD, *n* = 5), PD (*n* = 3), LBD (*n* = 2), and FTD (*n* = 1). Geographically, most studies were conducted in East Asia (*n* = 46), with additional contributions from Western Europe (*n* = 16), North America (*n* = 7), Southeast Asia (*n* = 5), and the Middle East (*n* = 1). In terms of study design, there were 41 cross‐sectional studies, 25 case–control studies, 6 longitudinal studies, and 6 randomized pilot trials. These pilot trials represent small‐scale, preliminary study designed to assess the feasibility, usability, and initial diagnostic performance of the device in a target population.

### 3.1 Principles and applications of NIRS in neuroimaging and cognitive assessment

NIRS operates on the principle that near‐infrared (NIR) light can penetrate human tissues with minimal interference. As NIR light travels, it undergoes absorption by chromophores, such as hemoglobin, or scattering within the tissue, with scattering occurring significantly more frequently.^20^ This mechanism forms the foundation of NIRS as a non‐invasive neuroimaging technique for monitoring brain activity.

Neurovascular coupling ensures that increased brain activity leads to heightened oxygen consumption and a corresponding rise in cerebral blood flow, which alters local concentrations of HbO_2_ and HbR.^21^ NIRS uses specific NIR wavelengths to measure these fluctuations, using distinct absorption properties of HbO_2_ and HbR to evaluate cerebral oxygenation.^22^ Spectral markers at 895 and 860 nm help differentiate AD, MCI, and healthy controls, with the 895 nm signal being particularly indicative of early‐stage MCI and the 860 nm signal becoming more prominent in later AD progression.^23^

The deployment of laser diodes through fiber optic bundles allows NIRS to target specific brain regions, such as the prefrontal and parietal cortices, using optodes strategically placed following the international EEG 10‐20 system. A secondary optode, positioned 4 cm laterally, enables a refined analysis of the reflected light, which is processed via a photomultiplier tube linked to a photon counter.^20^, ^22^ Raw intensity signals are converted into HbO_2_ and HbR concentrations using the modified Beer–Lambert Law, with standard correction techniques such as Butterworth filtering and detrending algorithms applied to reduce physiological noise and phase distortions.^24^, ^25^, ^26^

Functional NIRS (fNIRS), an advancement of NIRS with more optodes, enables extensive analysis of brain activity during cognitive tasks. The prefrontal cortex (PFC), occupying ≈ 29% of the total cerebral cortex volume, plays a central role in higher order cognitive functions, including attention, executive processing, memory, language, and visuospatial skills.^10^ It interacts with the hippocampus through functional connectivity (FC) networks, facilitating generalization of knowledge and supporting cognitive domains.^27^

### 3.2 Validation of NIRS as a reliable neuroimaging tool for cognitive assessment

NIRS has demonstrated strong consistency with well‐established neuroimaging techniques, including MRI, PET, EEG, and transcranial Doppler (TCD). NIRS measurements show a high spatial and temporal correlation with the blood oxygen level–dependent signal detected by MRI in brain network studies.^16^ Similarly, cerebral autoregulation (CA) measures derived from NIRS strongly correlate with TCD results, particularly during repeated sit‐to‐stand transitions in cognitively impaired patients, confirming its reliability in monitoring cerebrovascular function.^28^

Advancements in machine learning have further enhanced NIRS‐based diagnostics. Models incorporating olfactory‐stimulated oxygenation differences in the orbitofrontal cortex have demonstrated superior accuracy in distinguishing MCI and AD.^29^ Additionally, time‐resolved NIRS studies have shown significant positive correlations between MMSE scores and oxygen saturation (SO_2_) levels with a 91.5% accuracy in the left and right PFC,^30^ reinforcing its sensitivity in cognitive assessments. Convolutional neural network (CNN)–based NIRS models have also demonstrated > 89.57% accuracy in distinguishing MCI, surpassing the performance of the Korean version of the MoCA. Notably, the highest accuracy of 96.09% was achieved using the HbO_2_ slope measured from the left PFC within a 20 to 60 second time window.^31^ Similarly, multi‐dimensional NIRS has demonstrated strong classification accuracy for MCI, achieving 80.77% test accuracy, 76.92% sensitivity, and 83.33% precision.^32^ The NIRS index has further demonstrated high sensitivity (94%) and specificity (88%) in distinguishing MCI patients from healthy controls.^33^

Blood‐based NIRS has additionally emerged as a promising biomarker‐driven approach for AD detection. Multivariate classification models of blood‐based NIRS have achieved 92.8% accuracy, 87.5% sensitivity, and 96.1% specificity, highlighting its potential for minimally invasive diagnostics.^34^ Recent studies have also shown that NIRS can detect AD with a sensitivity of 0.76 and a specificity of 0.68. Additionally, when assessing the severity of AD using five or fewer measurement channels, NIRS achieved a sensitivity of 0.75 and a specificity of 0.72, indicating promising potential in low‐resource diagnostic settings.^35^ Studies indicate that SPECT achieves a sensitivity of 77% and specificity of 89% for diagnosing AD, whereas PET scans consistently demonstrate both sensitivity and specificity at 86%.^36^ When data are pooled across multiple studies, PET maintains an overall sensitivity of 86%, specificity of 84%, and accuracy of 85%. In comparison, pooled findings for SPECT report a sensitivity of 76% and specificity of 84%.^36^, ^37^ These diagnostic metrics align closely with the sensitivity and specificity scores reported for NIRS, supporting its role as a reliable, non‐invasive, and clinically relevant neuroimaging modality in AD assessment.

### 3.3 Application of NIRS in MCI and SCD

Among studies focusing on MCI and SCD, cross‐sectional designs were most commonly used (*n* = 36), followed by case–control (*n* = 9), longitudinal (*n* = 5), and randomized pilot trials (*n* = 3). Geographically, the majority of research was conducted in East Asia (*n* = 37), with further contributions from North America (*n* = 7), Southeast Asia (*n* = 4), and Western Europe (*n* = 5). MCI and SCD are widely recognized as early indicators that precede AD and other forms of dementia.^38^, ^39^, ^40^ Early‐stage cognitive impairment is characterized by declining function in the cerebral task‐positive network, accompanied by weakened FC among the frontal, temporal, and parietal lobes.^41^, ^42^ The PFC, particularly the dorsolateral region, plays a central role in memory processing, which is impacted in both amnestic MCI (aMCI) and SMD.^43^ Its hemodynamic response, measured through NIRS, provides a reliable region for assessing cognitive function.^14^, ^44^, ^45^ While normal aging increases prefrontal activation as a compensatory mechanism, MCI individuals experience greater task‐related declines and weakened prefrontal compensatory networks.^46^, ^47^, ^48^

NIRS assessments during working memory cognitive tasks, such as the N‐back Working Memory Task reveal significant disruptions in neurovascular coupling and FC, particularly in the left dorsolateral prefrontal cortex (LDLPFC), among individuals with MCI.^49^, ^50^ NIRS measures during visual memory span, verbal digit span task, and retrieval tasks also show significant lower HbO_2_ levels in the dorsolateral PFC of aMCI individuals but not in non‐aMCI (naMCI) individuals.^44^, ^51^, ^52^, ^53^ Complementing this, baseline NIRS findings during the Verbal Fluency Task also reveal distinct alterations in frontotemporal activation patterns that may serve as early indicators of cognitive decline but findings were not mirrored in the clock‐hand‐angle discrimination task.^54^, ^55^ Although overall Stroop task performance appears similar between individuals with MCI and healthy controls,^51^ task duration significantly impacts the observed neural responses. During the early stage (0–15 seconds), both MCI and healthy controls groups exhibit similar cortical activation. However, in the late stage (15–30 seconds), MCI individuals show compensatory increases in HbO_2_ levels, particularly in the ventrolateral PFC and parietal lobe, indicating enhanced neural engagement in response to sustained cognitive demand.^56^ On the other hand, naMCI individuals exhibit hyperactivation during Stroop tests in the right prefrontal region.^57^

In contrast, two‐back and Stroop tasks have also revealed a lack of activation in the ventrolateral PFC of MCI individuals.^58^ These contrasting findings may stem from the heterogeneous nature of MCI, which can vary by subtype and severity. Moreover, activation of the ventrolateral PFC depends on the integrity of its FC, contributing to the observed differences in brain activation patterns. Walking tasks have also shown no significant differences in overall FC between cognitively impaired and cognitively healthy groups.^59^ Additionally, the finger‐tapping task despite its cognitive demand has demonstrated limited discriminatory power in NIRS assessments of MCI with MCI individuals exhibiting sustained HbO_2_ levels, indicating that these task does not effectively distinguish between cognitive impairment and normal aging.^60^ The distinct nature of aMCI and naMCI necessitates targeted cognitive tests and PFC‐specific assessments for improved diagnostic accuracy. These findings further highlight the critical need for the deliberate selection of cognitive tasks that align with the specific neurophysiological changes targeted in NIRS assessments, thereby enhancing the sensitivity and precision of early‐stage diagnostic evaluations.

Compared to single tasks during NIRS assessments, which may not impose sufficient cognitive demand to clearly differentiate cognitively normal individuals from those with MCI, dual‐task paradigms have yielded a greater number of significant findings, enhancing the distinction between MCI and healthy controls.^48^, ^59^ Dual‐task results reveal significant intergroup differences in HbO_2_ values across all channels including those near the primary sensory, visuomotor, anterior PFC, and dorsolateral PFC regions among healthy young adult, cognitively normal, and MCI groups.^48^, ^61^ These differences may not be detectable with single‐task NIRS assessment. Additionally, during classification analysis, deep learning (CNN) models have significantly showed improved classification accuracy (90.62%) compared to linear models (76.7%).^62^ On the other hand, increasing task loads in a visual working memory task reveal neural compensation in MCI patients. At moderate task loads, hyperactivation in the PFC suggests preserved compensatory mechanisms, enabling sustained cognitive performance.^63^, ^64^, ^65^ As task difficulty rises, the MCI group continues to show elevated HbO_2_ levels, suggesting their ability to engage neural compensation under manageable cognitive demands.^64^, ^66^

Studies on FC further highlight disruptions in neural pathways as a core characteristic of cognitive impairment.^63^, ^65^, ^67^ Reduced interregional connectivity between the PFC and occipital lobes has been observed across multiple time intervals in the MCI group in resting state, indicating disrupted communication pathways in these key cognitive and visual processing areas.^10^, ^68^, ^69^ Unlike the widespread FC disruptions observed in dementia, early cognitive impairment presents localized abnormalities,^70^ offering a unique diagnostic opportunity. These altered connectivity patterns especially in MCI patients can also be demonstrated by graph‐based NIRS network analysis.^71^ The NIRS index, derived from logistic regression, has also proven valuable for assessing cognitive decline during working memory tasks.^33^ Integrating NIRS with the Game‐Based Intelligence Test (GBIT) further enhances MCI detection. By using HbO_2_ and HbR parameters, a transformed cubic polynomial function can be constructed, in which coefficients serve as key markers for identifying MCI.^6^

Time‐resolved NIRS (TRS) also offers a stress‐free evaluation method using a deep neural network for PFC assessment.^30^, ^72^ TRS enables rapid measurement of HbO_2_, HbR, total Hb, and oxygen saturation (SO_2_), which correlates strongly with MMSE scores, reinforcing its potential for cognitive screening.^30^, ^73^ Moreover, NIRS has been integrated into intervention studies, providing valuable insights into cognitive impairment progression and rehabilitation strategies.^47^, ^74^, ^75^, ^76^, ^77^, ^78^, ^79^ Importantly, HbO_2_ has proven to be a more reliable indicator of cortical activation than HbR due to its superior signal‐to‐noise ratio (SNR).^13^, ^32^, ^47^ This suggests that HbO_2_ in prefrontal hemodynamics is a more reliable indicator of neural activity in functional assessments when using NIRS.

Individuals with SCD perceive a decline in their cognitive abilities compared to their previous normal state, even though their symptoms do not yet meet the clinical criteria for MCI.^80^ NIRS integrated with machine learning techniques offers a powerful approach for analyzing PFC activation during cognitive tasks in such individuals. Subject‐wise cross‐validation has revealed that as cognitive function declines, the stability of prefrontal connectivity weakens. Individuals with SCD showed reduced interaction between the left and right regions of the PFC, with this decline in connectivity being less pronounced than in the MCI group compared to healthy controls. ^10^ Additionally, SCD patients exhibit a stronger FC pattern across frontal lobe channels during the resting state, suggesting more preserved brain network integrity. Furthermore, SCD patients demonstrate higher overall average HbO_2_ concentrations in the frontal lobe during verbal fluency tasks, reflecting a greater neural response to cognitive challenges compared to MCI patients.^81^ Notably, the dorsolateral PFC (DLPFC) and orbitofrontal cortex are the most affected regions in SCD and MCI.^10^

**FIGURE 2:**
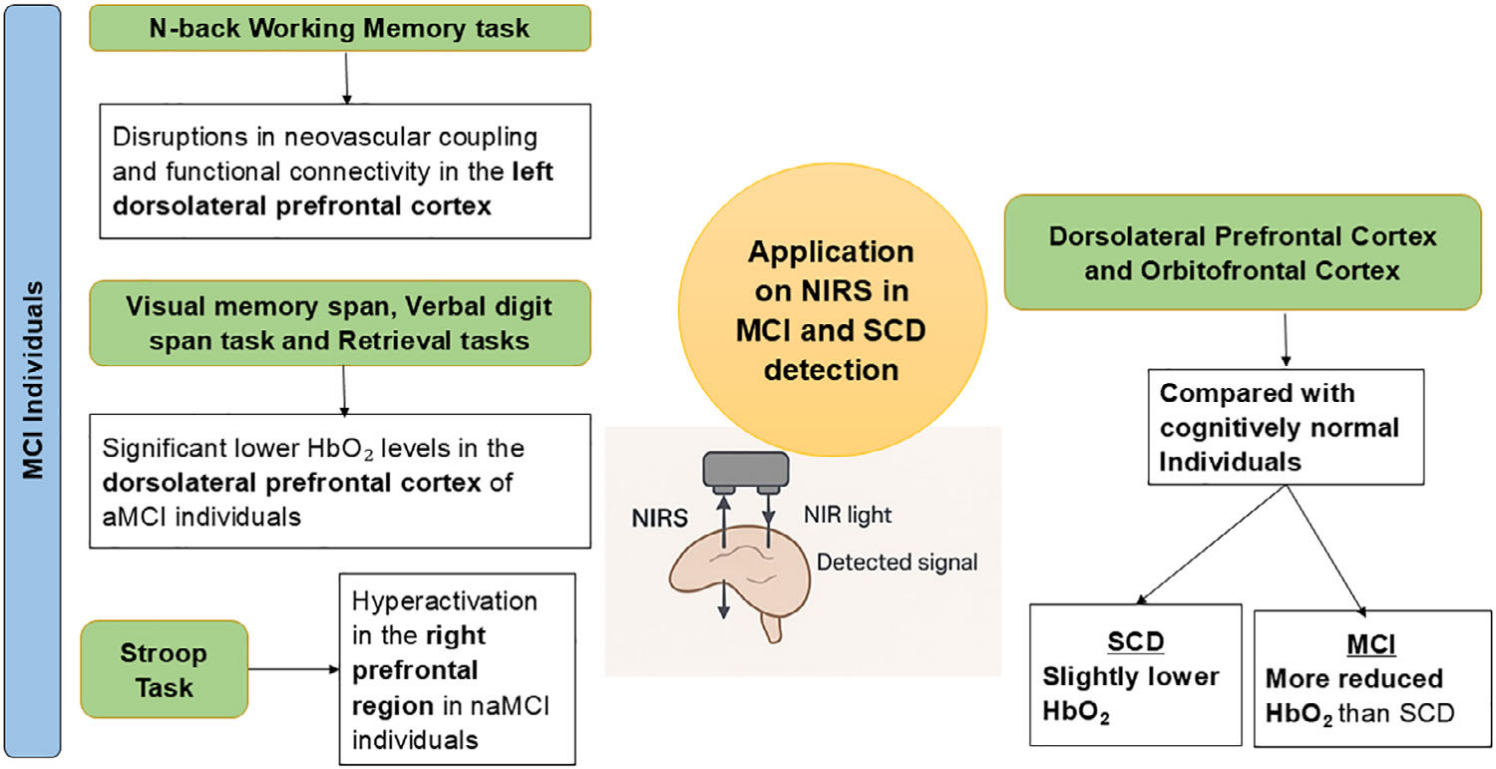
A visual overview of near‐infrared (NIR) spectroscopy (NIRS) applications in evaluating brain function among individuals with mild cognitive impairment (MCI) and subjective cognitive decline (SCD). It features several cognitive task‐based NIRS paradigms including the N‐back Working Memory Task, Verbal Digit Span, Visual Memory Span, Retrieval Tasks, and Stroop Task used to probe neurovascular dynamics. Findings highlight disrupted neurovascular coupling and reduced functional connectivity predominantly in the left dorsolateral prefrontal cortex (LDLPFC) in MCI, with reduced oxyhemoglobin (HbO_2_) levels in amnestic MCI (aMCI) and compensatory hyperactivation in the right prefrontal cortex observed in non‐aMCI (naMCI). In contrast, SCD individuals show milder impairments in the dorsolateral and orbitofrontal cortices, with slight HbO_2_ reductions compared to MCI individuals.

Elevated HbO_2_ levels have been observed in individuals with SCD compared to non‐SCD participants during both single‐ and dual‐task gait paradigms.^82^ In contrast, no significant differences in left PFC activation were found between healthy controls and individuals with subjective memory complaints (SMCs) during similar gait tasks.^83^ However, participants exhibiting more pronounced SMC demonstrated reduced HbO_2_ levels in the PFC during a visual memory span task.^44^ Collectively, these findings emphasize the utility of prefrontal hemodynamic responses as sensitive biomarkers for cognitive assessment, revealing a subtle yet progressive decline in neural efficiency associated with increasing memory‐related concerns. The distinction between SCD and MCI is crucial for early intervention, as SCD represents a subjective perception of cognitive decline without meeting clinical MCI criteria.^84^ Therefore, in‐depth FC analyses incorporating machine learning algorithms and graph theory, combined with HbO_2_ characteristics using NIRS during resting‐state and cognitive tasks, can facilitate differentiation between MCI, SCD, and cognitively healthy individuals.^26^, ^81^ A visual summary of the application of NIRS in assessing brain function in individuals with MCI and SCD is shown in Figure 2.

### 3.4 Application of NIRS in AD

Among studies investigating AD, case–control designs were most prevalent (*n* = 15), followed by cross‐sectional (*n* = 10), longitudinal (*n* = 2), and randomized pilot trials (*n* = 1). Geographically, research efforts were concentrated in East Asia (*n* = 11) and Western Europe (*n* = 11), with additional contributions from Southeast Asia (*n* = 4) and North America (*n* = 2). NIRS also offers an effective method for the early detection of AD and guiding personalized treatment strategies.^13^ The correlation between NIRS‐derived indicators such as activation levels, mean hemoglobin concentration, and FC of resting brain networks and clinical assessment scores suggests that NIRS has significant potential as a routine clinical tool for monitoring cognitive function, tracking AD progression, and evaluating rehabilitation outcomes.^13^, ^85^, ^86^

During cognitive tasks, NIRS enables precise identification of differences in brain activity patterns. Verbal fluency tests assess key cognitive functions, including executive function, processing speed, word retrieval, and memory.^18^ NIRS signals from the frontal region during verbal fluency tasks reveal distinct HbO_2_ response patterns between AD patients and healthy controls, with AD patients demonstrating reduced HbO_2_ concentrations in the dorsolateral prefrontal and superior temporal gyrus,^18^ premotor cortex, Broca's area, and prefrontal areas.^13^, ^85^ In contrast, evidence from a similar verbal fluency task study observed no significant activation differences in the PFC between mild AD and healthy controls.^87^ This discrepancy suggests that neurovascular alterations in early AD may be more subtle or variable across individuals. However, mixed phonemic and semantic verbal fluency tasks assessed with NIRS offer promising avenues for detecting preclinical AD stages by measuring interhemispheric FC trends during repeated cognitive trials.^88^ Additionally, AD patients exhibit increased activation in the left ventral PFC during positive emotional word processing, while increased activity in the right ventrolateral PFC is observed during neutral word conditions, reflecting distinct neural processing mechanisms.^89^

Moreover, resting‐state activation in bilateral prefrontal regions is notably lower in AD patients compared to healthy individuals, with the dorsolateral PFC showing the highest relative activity in healthy controls.^35^ Feature priority analysis highlights the LDLPFC as a critical region for distinguishing AD patients from cognitively healthy individuals. Subregional observations reveal that FC declines are most pronounced in the DLPFC, while connectivity in parietal areas remains relatively preserved and correlates positively with broader cognitive network integration.^13^, ^18^, ^65^, ^90^ These connectivity deficits may be associated with amyloid beta (Aβ) deposition, as evidenced by PET studies revealing amyloid accumulation in the frontal, parietal, temporal, and occipital cortices during the early stages of AD.^91^ Furthermore, diminished connectivity strength is predominantly observed in left prefrontal brain regions, potentially influenced by right‐handedness,^13^ an aspect that merits further investigation. Reduced left‐hemisphere activation during verbal fluency tasks further emphasizes the asymmetric progression of brain atrophy in AD.^18^

This increased unilateral hemisphere impact in AD is largely driven by the asymmetric progression of brain atrophy, which disrupts the normal asymmetries established during development.^92^ Age‐related cognitive decline leads to reduced hemispheric lateralization, yet NIRS in resting‐state verbal tasks consistently reveals lower interhemispheric FC in AD patients.^92^, ^93^ These disruptions, particularly affecting the premotor cortex, supplementary motor cortex, primary motor cortex, inferior parietal cortex, primary somatosensory cortex, and supramarginal gyrus, suggest that NIRS‐based assessment of interhemispheric connectivity may provide valuable insights into AD progression.^92^ Furthermore, increasing task load as a means to induce neural compensation in visual working memory tasks fails to produce substantial changes in bilateral PFC activation among mild AD patients. This pattern reflects diminished neural compensation mechanisms under cognitive demand, as prefrontal activation remains relatively stable with minimal signs of increased engagement, suggesting an impaired ability to recruit additional neural resources in response to task complexity.^64^

The reduction in HbO_2_ concentrations in the prefrontal lobe can also be assessed using NIRS through the sample entropy metric, particularly in tests evaluating visuospatial and short‐term memory functions.^94^ Among these assessments, the Clock Drawing Test, which evaluates visuo‐constructive and visuo‐spatial abilities, has demonstrated high reliability in distinguishing AD patients from healthy individuals, whereas the Digit Span Test and Corsi Block Tapping Test have not shown significant differentiation between groups.^95^ This reduction, possibly linked to vascular dysfunction, impairs local cerebral blood flow during cognitive processing, delaying expected fluctuations in oxygenation.^13^ Alternatively, AD‐related neurodegeneration may lead to functional reorganization, requiring compensatory blood flow adjustments in other brain regions to maintain function, thereby increasing overall oxygen consumption.^13^, ^96^

The integration of deep learning models with NIRS further enhances classification accuracy in AD diagnosis by identifying distinct changes in brain activation. Prodromal AD patients demonstrate reduced activation compared to healthy individuals and asymptomatic AD, highlighting early cognitive decline, although differentiation between asymptomatic AD and AD dementia remains challenging in resting‐state activation.^97^ Additionally, gradual declines in HbO_2_ concentration during verbal span tasks correspond with disease progression from MCI to severe AD.^98^ Advancements such as wavelet‐based motion artifact correction and orthogonal minimal spanning tree network binarization techniques improve the reliability of NIRS‐based connectivity assessments, providing robust insights into AD‐related network impairments.^99^

Olfactory dysfunction is another hallmark of AD, primarily attributed to plaque and tangle accumulation in the olfactory bulb and inner olfactory cortex, which play essential roles in memory formation.^100^ Olfactory‐stimulated NIRS reveals significant reductions in orbitofrontal cortex oxygenation levels, corresponding with cognitive performance scores and reinforcing the link between olfactory dysfunction and AD pathology.^101^ Machine learning–enhanced olfactory‐stimulated NIRS further improves classification accuracy in distinguishing MCI from AD.^29^

Beyond neuroimaging, NIRS applied to blood plasma samples presents a novel and minimally invasive technique for early AD detection. Blood serves as a rich biological medium due to the semi‐permeability of the blood–brain barrier, allowing molecular exchange between the brain and peripheral circulation, alongside the daily absorption of 500 mL of CSF into the bloodstream.^102^ Plasma is extracted through centrifugation at 2000 rpm for 10 minutes at 4°C, aliquoted, and stored at −80°C until spectroscopic analysis.^34^ Before testing, samples are thawed, applied to infrared‐reflective glass slides, and dried overnight at room temperature. Statistical analyses of plasma‐based NIRS data have identified significant spectral differences between healthy controls and AD patients, achieving 92.8% classification accuracy, 87.5% sensitivity, and 96.1% specificity.^34^ This method presents a highly precise and non‐invasive approach for detecting AD‐related blood signatures.

**FIGURE 3:**
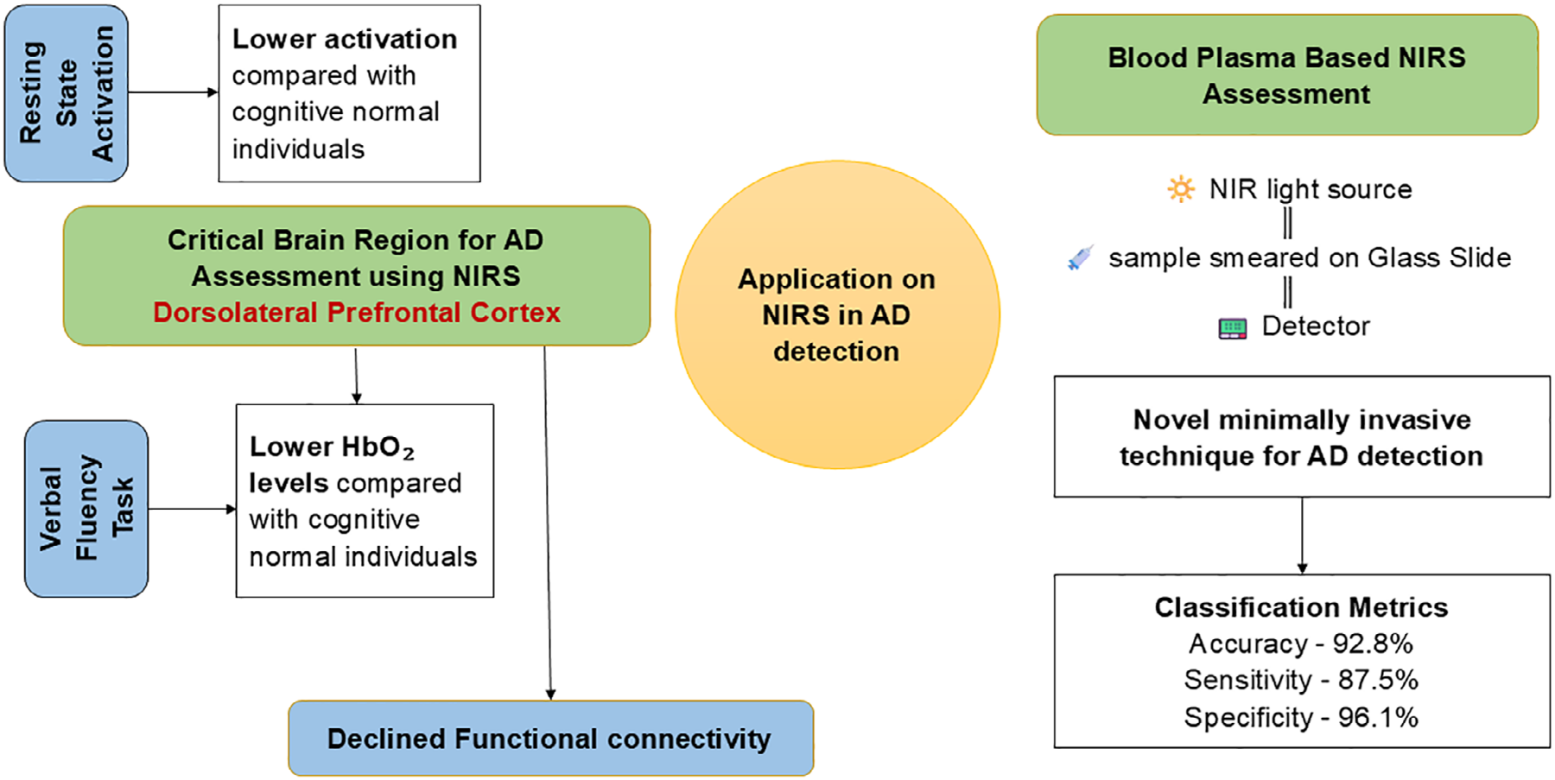
A visual illustration of near‐infrared (NIR) spectroscopy (NIRS) applications in detecting Alzheimer's disease (AD). The dorsolateral prefrontal cortex (DLPFC) represents a critical area of interest, highlighting reduced brain activation at rest, diminished oxygenated hemoglobin during verbal fluency tasks, and decline in functional connectivity in patients with AD. The infographic also showcases the emerging use of NIRS in blood plasma analysis for minimally invasive AD biomarker detection. A plasma‐based NIRS system presents with high diagnostic accuracy of 92.8% accuracy, sensitivity of 87.5%, and specificity of 96.1%. HbO_2_, oxyhemoglobin.

Overall, NIRS has proven capable of analyzing a wide range of complex neural and biochemical variables, demonstrating high specificity and sensitivity in AD detection (Figure 3). Its integration into routine clinical assessments may enhance diagnostic precision, facilitate disease monitoring, and improve therapeutic interventions for individuals with AD.

### 3.5 Application of NIRS in PD and LBD

Cognitive decline in PD affects various domains, including executive function, visuospatial skills, and memory. It is primarily characterized by deficits in executive processing, such as impaired conflict detection, reduced selective attention, and diminished inhibitory control.^103^ Whole‐head NIRS applied as an ecological monitoring tool for cortical activity also provides insights into neural activation patterns associated with PD progression. Early PD patients demonstrate increased activation in both the left and right secondary visual cortices, while individuals with mild PD show stronger activation in the right PFC, reflecting functional adaptations in response to disease severity.^104^ Furthermore, prefrontal activation progressively increases across all phases of obstacle crossing in PD patients, with significantly greater activation compared to healthy older adults, particularly during and after the crossing phase.^105^ Notably, PD patients display increased prefrontal activation when encountering unexpected obstacles, whereas activation levels remain comparable to healthy individuals during anticipated obstacle crossings.^105^ These findings suggest an increased reliance on prefrontal cortical engagement for motor planning and execution in PD, underscoring NIRS as a valuable tool for monitoring cerebral adaptations during dynamic movement. Moreover, HbO_2_ provides a more precise measure of task‐related cortical engagement compared to HbR between PD patients and healthy controls.

FC assessments using NIRS during the Stroop Color‐Word Test have revealed distinct neural processing differences among PD patients with MCI (PD‐MCI), those with normal cognition (PD‐NC), and healthy controls.^106^ PD‐MCI individuals exhibited increased regional strength measures (RSr and RSl) and global efficiency (GE) during the color‐word incongruent condition, with RSr reflecting the regional strength of the right PFC, emerging as a key predictive marker for distinguishing PD‐MCI from PD‐NC.^106^ The neural framework of inhibitory control operates within a right‐lateralized network involving the presupplementary motor area, inferior frontal gyrus, and subthalamic nucleus, reinforcing the critical role of right‐hemisphere neural structures in PD‐MCI.^107^ Attentional processing relies on two key brain networks: the ventral network, made up of right frontoparietal regions that help shift attention, and the dorsal network, which includes both sides of the frontoparietal cortex and supports sustained focus through top‐down control.^108^ Together, these systems highlight the right‐sided dominance of brain activity often seen in individuals with PD‐MCI.

LBD shares several clinical characteristics with AD, including impairments in memory, learning, orientation, comprehension, judgment, language, and visuospatial function. Increased Lewy body density contributes significantly to verbal fluency deterioration. Compared to AD patients, individuals with LBD exhibit superior verbal learning abilities but experience greater difficulty with verbal forgetting and recency effects, distinguishing them from AD in cognitive assessments.^109^ NIRS measurements indicate that LBD patients show higher HbO_2_ concentrations in the left temporal cortex, right DLPFC, and right temporal cortex during verbal fluency tasks than AD patients, suggesting improved cerebral oxygenation in these regions.^110^ Furthermore, integrating NIRS assessments with blood biomarkers enhances diagnostic accuracy, with LBD patients exhibiting higher α‐synuclein expression and lower Aβ42 levels compared to AD patients.^110^

### 3.6 Application of NIRS in FTD

The behavioral variant of FTD (bvFTD) is recognized as the most common form of frontotemporal lobar degeneration.^111^ fNIRS has been instrumental in identifying distinct cortical activation patterns associated with bvFTD during cognitive assessments such as the verbal fluency task. While AD typically elicits activation in the DLPFC and superior temporal gyrus, bvFTD is marked by increased activity in Broca's area located in the posterior portion of the left inferior frontal gyrus, suggesting potential compensatory mechanisms or alternative neural processing pathways in speech and language production.^18^ As Broca's area plays a central role in language formation, grammar, and sentence construction, its differential activation pattern may offer a distinctive biomarker for differentiating FTD from AD in clinical and research contexts.

### 3.7 Synergetic and future application of NIRS and other diagnostic platforms in dementia detection

Recent advancements in neuroimaging have underscored the utility of NIRS and multimodal techniques in detecting cerebral alterations associated with AD. Temporal stability in the coupling between cortical hemodynamic signals and CSF water signals varies across time windows and overlaps. Using a sliding window cross‐correlation approach, MRI‐compatible multiwavelength NIRS identifies marked differences in hemodynamic–CSF coupling patterns, particularly within the cardiac frequency band, between AD patients and healthy individuals.^17^ Healthy participants exhibit greater time‐domain variability in venous HbR‐CSF signal pairings (HbR‐CSF, dHbR‐CSF), whereas AD patients demonstrate more uniform coupling,^17^ possibly due to cerebrovascular stiffness and impaired CSF regulation resulting from vascular pathology and tissue atrophy.^112^, ^113^

To further elucidate neurophysiological dysfunction in AD, integrative approaches combining EEG and NIRS have proven effective. EEG offers high temporal resolution of cortical electrical signals, while NIRS provides hemodynamic insights, and their synchronization achieved through time‐locked triggers and standardized cap configurations enables simultaneous monitoring of neural and vascular responses.^114^ When applied during cognitive tasks such as the digit verbal span, this EEG–NIRS fusion captures weakened cortical connectivity in AD, with EEG features localizing primarily to the left parietal region and NIRS features in the right frontal cortex.^115^, ^116^

Entropy‐based analyses further strengthen this framework by using sample entropy to evaluate neural signal unpredictability and conditional entropy to assess the interdependence between EEG and NIRS signals. These complexity metrics have demonstrated superior classification accuracy in working memory assessments especially during the recall phase of the Rey–Osterrieth Complex Figure test compared to unimodal approaches.^117^ Moreover, hybrid EEG–NIRS models outperform EEG or NIRS alone in differentiating AD stages.^116^

Broadening this integrative scope, multimodal imaging incorporating EEG, NIRS, electrocardiography (ECG), and respiratory effort monitoring reveals additional physiological dysregulation in AD. Findings show altered neurovascular coherence, reduced brain oxygenation power, and impaired autonomic responses, including decreased instantaneous heart rate (IHR)–NIRS coherence and disrupted respiratory–NIRS relationships.^118^ These anomalies are particularly evident across neurogenic, myogenic, and endothelial frequency bands, reinforcing the value of multi‐system assessments in tracking AD progression.

CA, the mechanism stabilizing cerebral blood flow through arteriolar response to blood pressure changes, also exhibits changes in AD.^28^ CA works alongside neurovascular coupling, which increases local blood flow based on neural activity and is influenced by systemic and cerebral CO_2_ levels.^119^ While static CA eludes traditional imaging modalities, dynamic CA evaluated via TCD or NIRS can reveal real‐time vascular reactivity. NIRS‐ and TCD‐derived CA measures demonstrate stronger correlations during sit‐to‐stand transitions than during resting states in cognitively impaired individuals, suggesting that dynamic tasks enhance sensitivity for detecting cerebrovascular dysfunction associated with AD.^28^, ^120^ Together, these findings highlight the expanding role of NIRS, alone and in multimodal integration, as a powerful tool for non‐invasive investigation of cortical connectivity, vascular dynamics, and autonomic regulation in AD, MCI, and other NDDs.

Despite the promising applications of NIRS in cognitive assessment and NDD detection, NIRS faces several limitations that affect its reliability and interpretation. Instrumental challenges, including low spatial resolution, and poor SNR, which can result from inadequate hardware choices or set‐up, can lead to issues like cross‐talk and contamination from superficial tissues.^21^ Methodologically, the complexity of data processing poses another hurdle, as consistent preprocessing and reporting standards are lacking across studies.^24^ This complicates comparisons and undermines reproducibility. Moreover, physiological artifacts such as heartbeat and breathing can interfere with brain oxygen signals, further distorting results.^69^ Future research should explore correction techniques such as adaptive filtering or multimodal data fusion to minimize physiological noise and improve signal precision. Integrating machine learning and automation offers promise for enhancing data clarity, but the field still requires greater standardization and refinement to overcome these challenges. Standardization of protocols would enhance reproducibility and improve cross‐study validation, allowing for more reliable conclusions about NIRS‐based biomarkers for cognitive assessment. Additionally, systemic physiological factors including metabolic disorders, vascular aging, blood oxygenation levels, and cerebrovascular reactivity, can affect hemoglobin absorption and scattering properties, potentially influencing NIRS readings and introducing confounding variables.

Moving forward, large‐scale validation studies, incorporating multimodal imaging approaches (EEG fNIRS, PET fNIRS) and longitudinal monitoring, will be essential for establishing NIRS as a routine diagnostic modality in NDD detection and cognitive function assessment. Additionally, NIRS is particularly well suited for use in low‐resource settings due to its non‐invasive nature, portability, and cost effectiveness. Unlike traditional imaging techniques like MRI or CT scans, NIRS requires minimal infrastructure and can be easily set up in various environments, such as clinics. NIRS systems cost between $24,000 and $500,000, making them significantly more affordable than conventional neuroimaging methods. By comparison, MRI scanners can cost ≈ $1.4 to $1.5 million, based on reports from the UK and Belgium.^121^ These comparisons underscore the economic feasibility of NIRS, especially in resource‐limited settings in which access to high‐cost neuroimaging infrastructure remains a challenge. Also, its ability to provide real‐time monitoring of brain activity without the need for extensive training makes it accessible for health‐care workers in these regions. Moreover, NIRS is less sensitive to motion, allowing for more naturalistic testing conditions, which is crucial for accurately assessing cognitive function in diverse populations. These features collectively enhance the feasibility and effectiveness of early dementia detection in resource‐limited areas.

## 4 CONCLUSION

NIRS is a powerful, non‐invasive tool for assessing cognition across MCI, SCD, and various neurodegenerative conditions like AD, PD, LBD, and FTD. Critically, NIRS accurately detects altered neurovascular coupling, FC deficits, and hemodynamic responses during cognitive tasks, enhancing early diagnosis and differentiation among disease subtypes, a significant advantage in resource‐limited settings in which advanced neuroimaging is often unavailable or prohibitively expensive.

Multimodal approaches, combining NIRS with techniques like EEG and TCD, significantly improve diagnostic accuracy and expand clinical utility, offering more comprehensive assessments with potentially fewer logistical hurdles than traditional methods. Additionally, blood‐based NIRS shows promise for AD detection, reinforcing its potential in biomarker development and providing a highly accessible screening method for populations with limited access to specialized clinics. As research progresses, NIRS continues to evolve as a crucial tool for both diagnosis and monitoring, supporting early intervention and deepening our understanding of cognitive decline. Future research should prioritize longitudinal studies to track NIRS marker progression across disease stages. Furthermore, investigating NIRS findings in diverse ethnic groups, particularly underrepresented populations, is essential for a more inclusive understanding of neurological conditions and ensuring the equitable application of NIRS technology especially in low‐resource environments.

## AUTHOR CONTRIBUTIONS

*Conceptualization*: George Nkrumah Osei, Ansumana Bockarie, Peter Osei‐Wusu Adueming and David Larbi Simpong. *Methodology and Investigation*: George Nkrumah Osei, Ansumana Bockarie, Albert Akpalu, Maxwell Hubert Antwi, Foster Appiah, Linus Kpelle, David Mawutor Donkor and David Larbi Simpong. *Supervision*: Ansumana Bockarie, Peter Osei‐Wusu Adueming and David Larbi Simpong. *Writing – original draft*: George Nkrumah Osei, Maxwell Hubert Antwi, Foster Appiah, Linus Kpelle, David Mawutor Donkor and David Larbi Simpong. All authors revised the manuscript critically for important intellectual content. All authors read and agreed with the final manuscript.

## CONFLICT OF INTEREST STATEMENT

The authors declare no conflicts of interest. Author disclosures are available in the Supporting Information.

## Supporting information



Supporting Information

Supporting Information

Supporting Information

## Data Availability

No data were used for the research described in the article.
