# Supplementary figures and images for: Diagnostic potential of near‐infrared spectroscopy in mild cognitive impairment and neurodegenerative disorders: Implications for resource‐limited settings
Source: Alzheimers Dement. 2025 Oct 8;21(10):e70769. doi: 10.1002/alz.70769 (PMC12505193; doi:10.1002/alz.70769)

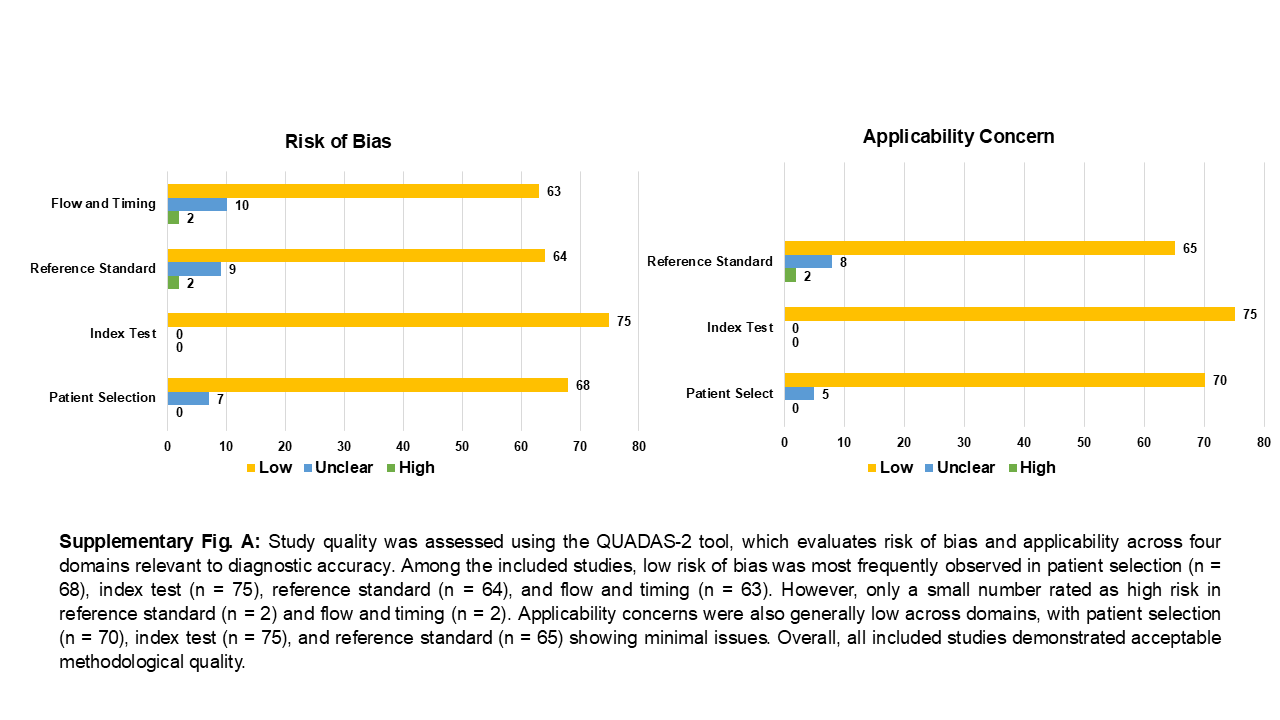

Supplement: Supplementary file 1 — Supporting Information [file ALZ-21-e70769-s001.tif]
