# Supplementary material for: Diagnostic potential of near‐infrared spectroscopy in mild cognitive impairment and neurodegenerative disorders: Implications for resource‐limited settings
Source: Alzheimers Dement. 2025 Oct 8;21(10):e70769. doi: 10.1002/alz.70769 (PMC12505193; doi:10.1002/alz.70769)
Supplement: Supplementary file 3 — Supporting Information [file ALZ-21-e70769-s002.docx]

**Search strategy**

***PubMed***

('near-infrared spectroscopy' OR 'NIRS') AND ('diagnosis' OR 'detection' OR 'management') AND ('neurodegenerative disease' OR 'cognitive impairment' OR 'Alzheimer's Disease')

***Scopus***

( near-infrared AND spectroscopy OR 'nirs' ) AND ( 'diagnosis' OR 'detection' OR 'management' ) AND ( 'neurodegenerative disease' OR 'cognitive AND impairment' OR 'alzheimer's AND disease' )

***Google Scholar (Advanced Search):***

**with all of the words:** near infrared spectroscopy diagnosis detection management neurodegenerative disease cognitive impairment Alzheimer's Disease

**with the exact phrase:** near infrared spectroscopy

**with at least one of the words:** "near infrared" spectroscopy

**where my words occur:** anywhere in the article
